# Supplementary material for: Vitamin D Modified DSS-Induced Colitis in Mice via STING Signaling Pathway
Source: Biology (Basel). 2025 Jun 18;14(6):715. doi: 10.3390/biology14060715 (PMC12190092; doi:10.3390/biology14060715)
Supplement: Supplementary file 1 [file biology-14-00715-s001.zip › Supplementary Materials S1: 16s-report of gut microbiota/Groups/alpha_rarefaction/observed_otus_rarefaction_groups.pdf]

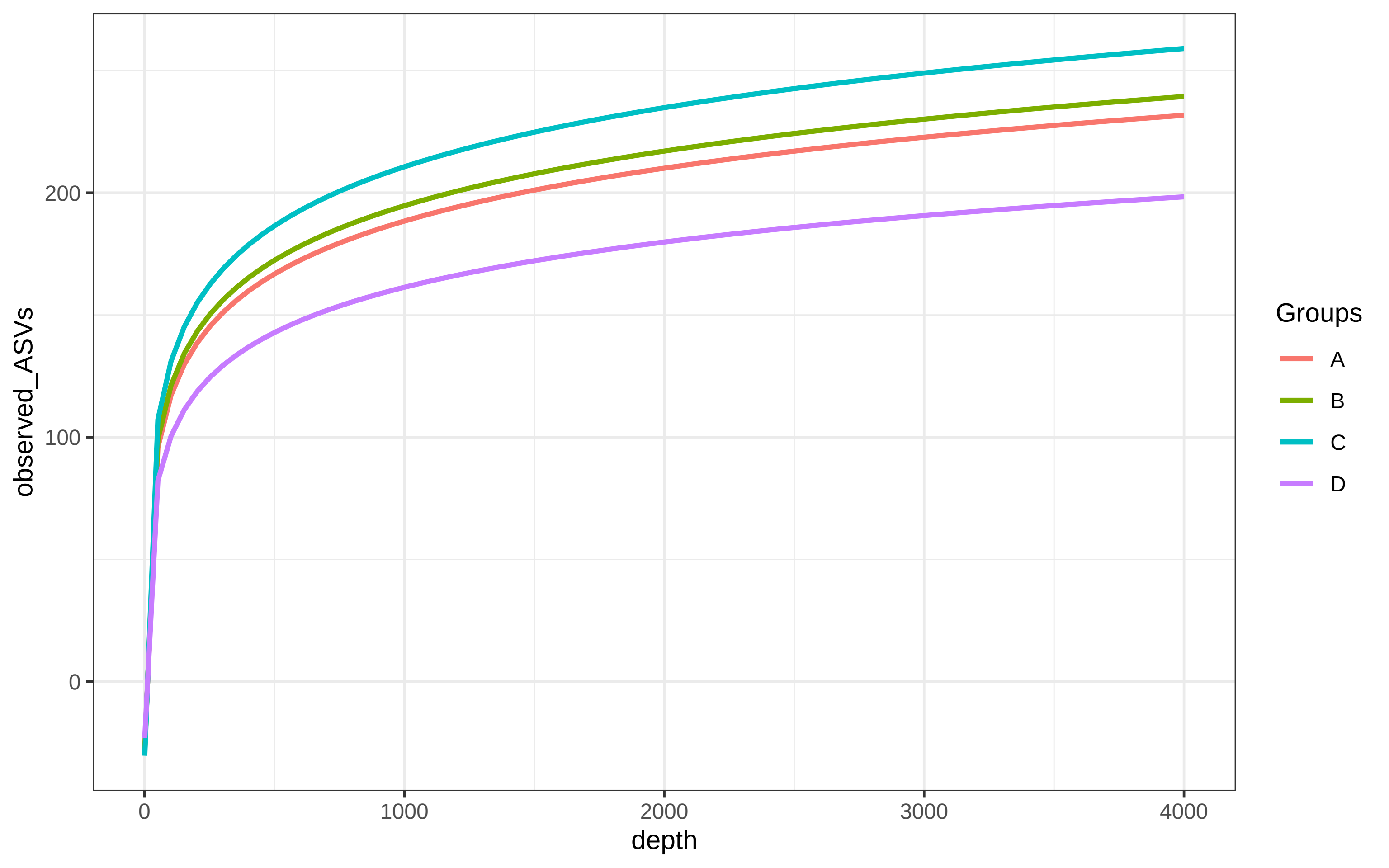

observed_ASVs
200
100
0
0
1000
2000
3000
4000
depth
Groups
A
B
C
D
